# Birth imprinting effects on the antibody responses of H7N9 patients from 2013-2018 in China

**DOI:** 10.1038/s43856-026-01554-1

**Published:** 2026-04-09

**Authors:** Xia Lin, Wenda Guan, Liping Chen, Yin Su, Zaolan Liang, Cheng Xiao, Yaqin Bai, Hui Lei, Fangmei Lin, Zhenting Huang, Isaac Cheuk Long Chow, Bingyi Yang, Tim K. Tsang, Yunceng Weng, Nisha Bhandari, Raghavan Varadarajan, Malik Peiris, Benjamin Cowling, Tang Li, Maureen McGargill, Paul G. Thomas, Nanshan Zhong, Richard Webby, Zifeng Yang, Mark Zanin, Sook-San Wong

**Affiliations:** 1 Guangzhou Medical University, Guangzhou, China; 2 State Key Laboratory of Respiratory Disease, National Clinical Research Center for Respiratory Disease, Guangzhou Institute of Respiratory Health, The First Affiliated Hospital of Guangzhou Medical University, Guangzhou, China; 3 HKU-Pasteur Research Pole, School of Public Health, LKS Faculty of Medicine, The University of Hong Kong, Hong Kong SAR, China; 4 Department of Biostatistics, St. Jude Children’s Research Hospital, Memphis, TN USA; 5 School of Public Health, LKS Faculty of Medicine, The University of Hong Kong, Hong Kong SAR, China; 6 Centre for Immunology & Infection, Hong Kong SAR, China; 7 WHO Collaborating Centre for Infectious Disease Epidemiology and Control, School of Public Health, LKS Faculty of Medicine, The University of Hong Kong, Hong Kong SAR, China; 8 Molecular Biophysics Unit, Indian Institute of Science, Bangalore, India; 9 Department of Immunology, St Jude Children’s Research Hospital, Memphis, TN USA; 10 Program in Immunology and Vaccine Development, Vaccine and Infectious Disease Division, Fred Hutchinson Cancer Center, Seattle, WA USA; 11 Department of Host-Microbe Interactions, St. Jude Children’s Research Hospital, Memphis, TN USA

**Keywords:** Influenza virus, Immunological memory

## Abstract

**Background:**

There is an urgent need to understand the immune correlates of protection against avian influenza viruses (AIV), where pre-existing immunity may be limited.

**Methods:**

Here, we characterized the antibody response in 12 severely ill A(H7N9) patients and examined its association with early-life imprinting and clinical outcome.

**Results:**

We find that A(H7N9) patients imprinted with A(H2N2) during early life show minimal H7-IgM and a rapid IgG response across diverse hemagglutinin subtypes. They also have more high avidity H7-antibodies compared to older or younger patients. Early antibody titers against seasonal H1, H3, and conserved stalk domains trend negatively with clinical severity in A(H7N9) infection, while an inverse pattern is observed following severe A(H1N1) infection, potentially suggesting a different mechanism of immune regulation between seasonal and avian influenza virus infections.

**Conclusions:**

These data provide direct serological evidence that birth imprinting profoundly shapes the humoral immune landscape during zoonotic influenza infection and may influence subsequent disease outcome.

## Plain language summary

This study aimed to examine how a person’s first flu infection in early life affects their immune response to avian flu later in life. We analyzed the immune responses of 12 severely ill patients and found that the first flu virus a person encounters in early life shapes their defense decades later. Patients whose first childhood flu was a strain known as H2N2 developed faster, stronger, and more specific immune response against a different strain, known as H7N9. Also, patients with a stronger immune response against common seasonal flu tended to have milder avian flu illness. This shows that early immune memory affects flu severity later in life. This understanding may help identify high-risk groups during outbreaks and improve age-specific vaccination strategies.

## Abstract (web summary)

Lin et al. investigate the immune response in severely ill H7N9 patients and its link to early-life influenza imprinting. They find that individuals first exposed to H2N2 develop faster, higher-avidity cross-reactive antibodies and tend to experience less severe illness.

## Introduction

To date, over 2884 human infections caused by 16 different AIV subtypes have been reported to the World Health Organization^1^. Of these numbers, A(H5N1) and A(H7N9) subtypes account for 33% and 54% of the total cases with case fatality rates approximating 50% and 39%^2^, respectively. Although infections can sometimes cause mild disease, severe infections lead to acute respiratory distress and pneumonia, with death often due to multi-organ failures^3^. Notably, infections by these two subtypes also appear to have distinct age susceptibility profiles. For example, 80% of A(H5N1) cases were in those younger than 35 years old while the mean age of A(H7N9) cases was 55 years of age^4^.

A proposed hypothesis to account for this age-specific morbidity is the impact of early-life immune imprinting by the phylogenetically-related influenza hemagglutinin (HA) protein^5^. The influenza A(HA) is classified into two phylogroups; H1, H2 and H5 subtypes belong to Group 1, while H3 and H7 belong to Group 2^6^. Exposure during early life to A(H1) or A(H2) subtypes, which circulated before 1957 and between 1957 and1968, respectively, were predicted to protect against severe A(H5N1) infections, whereas first exposure to A(H3), which circulated after 1968, protected against severe A(H7N9) infections. Whilst the serological evidence for imprinting reducing the risks of infection has been described for seasonal influenza^5,7,8^, evidence on how this phenomenon impacts antibody responses with AIV infection and whether it impacts disease outcome is lacking^5^. Here, to better understand the immune correlates of protection to AIV, we investigated the antibody profile of patients infected with the A(H7N9) AIV, within the context of immune imprinting and examined its impact on clinical outcome. We compared these responses to severely ill patients infected with seasonal A(H1N1) virus. Our findings provide direct serological evidence that birth imprinting shapes the magnitude, quality, and breadth of the antibody response to H7N9 infection, with evidence that imprinting-associated pre-existing immunity may modulate disease severity, highlighting the critical role of birth history in shaping humoral immunity to avian influenza viruses.

## Methods

### Ethics approval

This study was approved by the Ethics Committee of the First Affiliated Hospital of Guangzhou Medical University (reference number 2016-78). Written informed consent was obtained from all adult participants and from parents or guardians of minor participants included in the study.

### Serum samples

A total of 16A(H7N9) infected patients from laboratory confirmed, admitted between 2013 and 2018 were used in this study. Sera samples collected prior to 2017 were banked sera from patients admitted to the First Affiliated Hospital of Guangzhou Medical University, a referral hospital that specializes in respiratory diseases, while samples collected after 2017 were collected as part of a clinical study in different hospitals. Serum samples were stored at −80 °C between collection and immunological analyses. The patients were aged between 17 and 74 years old. Paired sera used in this study were collected at two intervals: the early sera was the earliest available sample, collected between days 9 and 18 post illness onset, and the late sera was collected at least 14 days later, between days 19 and 39. Details are summarized in Supplemental Table 1. For comparisons, sera were collected from 11A(H1N1) infected patients hospitalized at the First Affiliated Hospital of Guangzhou Medical University at early and late stage of infection as showed in Supplemental Table 2.

### Virus

Reverse genetics (rg) viruses used for the serology assays were propagated in 10-day-old embryonated chicken eggs. rgH7N1 viruses were generated using the seven genes of A/Puerto Rico/8/1934 (H1N1) virus but bearing the H7 HA gene derived from either A/Anhui/1/2013 (H7N9) (H7.AN13) or A/Qingyuan/GIRD01/2017 (H7N9) (H7.QY17). rgH6N9 viruses were generated using the mismatched H6 from A/teal/Hong Kong/W312/1997 (H6N1) with N9 gene derived from either AN13 or QY17. N1 NA gene of rgH6N1 viruses were from A/California/07/2009 (H1N1) and A/Michigan/45/2015 (H1N1).

### Hemagglutination inhibition assay (HI)

All the serum samples were treated with receptor-destroying enzyme (Denka Seiken, Tokyo, Japan) for 18 h and heat-inactivated at 56 °C for 30 min. Samples were diluted starting at 1:10 (v/v) in phosphate-buffered saline (PBS), followed by serial two-fold dilutions. HAI assay was tested with 1% horse red blood cells in V-bottom 96-well plates according to World Health Organization guidelines (https://apps.who.int/iris/handle/10665/44518).

### Enzyme-linked immunosorbent assay (ELISA)

Recombinantly produced and purified HA (Sino Biological Inc) at 0.5 µg/ml was coated onto 96-well plates overnight. After washing 3 times with PBST and blocking with PBST containing 3% FBS, sera were serially diluted and added to the plates starting at 1:200 for 2 h at 37 °C. Plates were washed and incubated for 1 h with horseradish peroxidase (HRP)-conjugated secondary antibodies (Bioss) as follows: anti-human IgG (H + L) (bs-0297G-HRP, 1:10000), anti-human IgA (H + L) (bs-0360G-HRP, 1:2000), and anti-human IgM (H + L) (bs-0345R-HRP, 1:2000). The reactions were terminated by hydrogen sulfate and the absorbance was read at 450 nm. Endpoint titers were defined as the last serum dilution that gave a positive/negative optical density readout ratio of >2. The area under the curve (AUC) of the ELISA response determined for Group 1 and Group 2 HA was calculated by GraphPad Prism (version 9) and IgG total AUC was the sum of Group 1 and Group 2 AUC.

### Microneutralization assay (MN)

Serum was diluted starting at 1:10 and mixed with 100 Tissue Culture Infectious Dose 50 (TCID_50_) of virus. After incubated for 2 h at 37 °C, Madin-Darby Canine Kidney (MDCK) cells were added in each well and incubated at 37 °C for 18 h. MDCK cells were kindly provided by Dr. Song Wenjun (Guangzhou Medical University, China). MN titer was determined as the reciprocal dilution that inhibited virus growth by an ELISA-readout method.

### Determination of IgG avidity

The IgG avidity index (AI) was determined essentially by the ELISA protocol with the addition of different concentration of urea. Sera was diluted to an expected absorbance of 1.0. After incubation of the diluted sera (at an expected absorbance of 1.0) with the antigen-coated wells, the wells were emptied and washed to remove unbound antibodies. Then, 4-8 molarity (M) urea or PBS was added for 30 min at room temperature before the detection step. The AI, expressed as a percentage (%), was the ratio of the optical density (OD) of urea washed samples and the OD of the samples not washed with urea (AI(%) = (OD of antigen with urea wash–OD of control with urea wash)÷(OD of antigen without urea wash–OD of control without urea wash)).

### Enzyme-linked lectin assay

Neuraminidase inhibition (NI) sera antibody titers were determined by enzyme-linked lectin assay (ELLA) using rg viruses expressing the NA of the target strains and a mismatched HA (H6). In brief, 96-well plates were coated with fetuin at 4 °C overnight. Sera samples were heat inactivated and serially diluted from 1:10 to 1:5120 and mixture of virus and sera was incubated for 18 h in fetuin-coated plates. Plates were washed with three times PBST and incubated with PNA-HRP for 2 h. The NI titers were measured from the serum dilution that yielded 50% inhibition of the virus control.

### Imprinting probability calculation

We reconstructed the birth cohort-specific imprinting probability (subtype of first influenza virus infection) using historical frequencies of seasonal influenza subtype, using the method as previously described^5,9^. This was based on influenza surveillance data from sequence data from GISAID and a previous age-seroprevalence study that suggested that all children were infected by influenza at least once by age 12. Based on a truncated geometric model, the annual infection probability was estimated to be 0.28^5^. Hence, based on the proportion of infections that were A(H1N1), A(H2N2) or A(H3N2) at each year informed by global surveillance data, the probability that the first influenza virus infection was subtype *v* (*p*_*v*_) was ${\sum }_{u=1}^{12}0.28{\left(1-0.28\right)}^{u-1}* {v}_{u}$, where ${v}_{u}$ was the proportion of infections that were subtype *v* in year *u* for *v* equal to 1 (H1N1), 2 (H2N2) and 3 (H3N2).

### Murray score calculation

The Murray score is the average of four criteria for an expanded definition of the acute respiratory distress syndrome, including chest radiographic findings, hypoxemia, level of positive end-expiratory and respiratory system compliance^10^. The calculation of the Murray score at different days after symptom onset in A(H7N9) and A(H1N1) patients was shown in Supplemental Data 1 and2.

### Statistics and reproducibility

Correlation analyses were generated by corrplot package (https://github.com/taiyun/corrplot) by using Pearson’s method in R (version 4.2.2), with the p-values adjusted by controlling for the False Discovery Rate using the Benjamini-Hochberg method. Heatmap analysis were used log_2_ transform end point titers and generated by “ComplexHeatmap” package in R (version 411 4.0.5). Data were analyzed by using GraphPad Prism version 9. Statistical significance between paired samples were determined by the two-sided Wilcoxon matched-pairs signed rank test and while differences between groups were determined by Mann-Whitney U Test. Statistical significance among three or more groups were determined by non-parametric Kruskal-Wallis Test, then the pairwise comparisons with significant *p*-values, *, *p* < 0.05; **, *p* < 0.01; ***, *p* < 0.001; and ****, *p* < 0.0001. The sample size for each experiment was detailed in the corresponding figure legend.

## Results

**Fig. 1:**
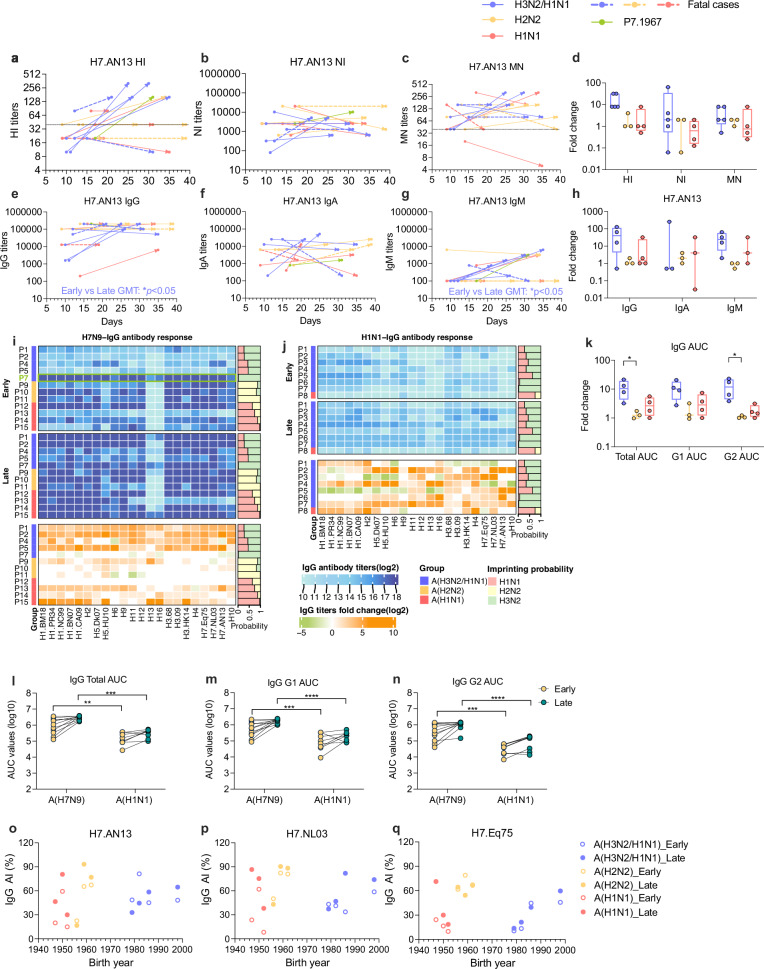
Immune imprinting-dependent antibody response patterns in A(H7N9) and A(H1N1) influenza infections. **a** Hemagglutination inhibition (HI) **b** neuraminidase inhibition (NI) antibody titers **c** microneutralization (MN) assays **d** HI, NI, and MN antibody fold-change in paired sera, **e**–**g**, H7.AN13-specific IgG, IgA, and IgM titers, and **h** binding antibody fold-change in paired sera against A/Anhui/1/2013 (A(H7N9) (H7.AN13) in A(H7N9) patients in three imprint groups; blue: A(H3N2/H1N1), *n* = 5, yellow: A(H2N2), *n* = 3, and red: A(H1N1), *n* = 4. P7 is colored in green while fatal cases are indicated with a dashed line. **i**, **j** Heatmap of IgG titers in A(H7N9) and A(H1N1) patients against both Group 1 (G1) and Group 2 (G2) HA as measured by ELISA. Fold changes in paired sera against G1 and G2 HA were shown in the lower panel. **k** Fold change in total HA, G1, and G2 area under curve (AUC) values between early and late sera of A(H7N9) patients from the different imprint groups. **l**–**n** AUC values for the early and late stage of A(H7N9) and A(H1N1) patients against IgG total, G1 and G2 HA. **o**–**q** The IgG avidity index (AI%) after 7 M urea wash against H7.AN13, A/Netherlands/219/03 (H7.NL03) and A/equine/Kentucky/1a/1975 (H7.Eq75) based on year of birth and colored according to imprint probability. Statistical significance between early and late antibody titers from each imprint group in (**a**, **b**, **d**–**f**) was analyzed by two-sided paired t-test on log₂-transformed titers (In A(H3N2/H1N1) group, *p = 0.028 for IgG, *p = 0.034 for IgM). Differences between A(H7N9) and A(H1N1) in (**k**, **m**) were analyzed by two-sided Mann-Whitney U test. In A(H7N9) vs. A(H1N1) comparison, all differences were statistically significant: IgG Total **p = 0.0063 (Early), ***p = 0.00012 (Late); IgG G1 ***p = 0.00061 (Early), ****p < 0.0001 (Late); IgG G2 ***p = 0.00021 (Early), ****p < 0.0001 (Late). Differences among three groups in (**d**, **h**, **k**) were determined by non-parametric Kruskal-Wallis Test, then the pairwise comparisons with significant *p*-values (A(H3N2/H1N1) vs. A(H2N2) for IgG Total (*p = 0.0372), G2 (*p = 0.025)). Dotted lines; HI titer = 1:40, MN titer = 1:40.

The outbreaks of A(H7N9) occurred over five waves in mainland China between 2013 and 2018, resulting in a total of 1568 cases^1^. We collected paired serum samples from 16A(H7N9) patients admitted to five hospitals in Southern China between 2013 and 2018 (Supplemental Table 1). Patients, with ages ranging from 17 to 74, were hospitalized between 9 and 18 days after illness onset, with a mean hospitalization and ICU stay duration of 44 (range; 20–101) and 33 (range; 12–84) days, respectively. Four patients (25%), including a pregnant woman, died during hospitalization. For subsequent analyses, we concentrated on antibody responses in the twelve survivors as the skewed immune responses in the fatal cases (Fig. 1 and Supplementary Fig. 1, dashed line) may lead to confounding results^11^. The first samples were collected upon hospital admission and second samples collected at least 14 days later. To study the impact of immune imprinting, we analyzed the patients antibody responses based on their probable birth imprinting history (see Method)^9^; mixed A(H3N2/H1N1) (born between 1967 and 1998, *n* = 5), A(H2N2) (born between 1956 and 1959, *n* = 3) and A(H1N1) (born in or before 1953, *n* = 4).

We first assayed for A(H7N9) specific antibody responses to A/Anhui/1/2013 (H7N9) (AN13) by HA (HI), neuraminidase-inhibition (NI) and microneutralization (MN) assays (Fig. 1a–d), which detect antibodies capable of inhibiting the receptor-binding (HI and MN) and release functions of the virus (NI), respectively. A/Anhui/1/2013 (H7N9) (AN13), a low pathogenic avian influenza A(H7N9) virus isolated during the first wave, is considered a prototypical strain of the first four waves of A(H7N9) viruses^12^. Of those imprinted with mixed A(H3N2/H1N1), 100% (5/5) seroconverted (HI titer fold change ≥4) to AN13, while only 33% (1/3) and 25% (1/4) seroconverted in the A(H2N2) and A(H1N1) groups, respectively (Fig. 1d). In contrast, the NI-antibody titers were much higher across groups and fold-changes were variable, resulting in no statistically significant differences in NI-antibody responses across the three imprinting groups (Fig. 1b, d). MN assay seroconversion rates were 40% (2/5) for A(H3N2/H1N1), 0% (0/3) for A(H2N2), and 25% (1/4) for A(H1N1) (Fig. 1c, d).

To assess changes to binding antibodies, we assayed H7-specific IgG, IgA, and IgM in early and late sera to AN13 (Fig. 1e–h) by ELISA. Except for P7, which already had high H7.AN13-IgG at admission (Fig. 1e), other patients imprinted with A(H3N2/H1N1) had lower titers. These patients (excluding P7) showed significant increases in their late sera IgG and IgM titers to H7.AN13 compared to other groups (Early vs Late GMT, *p* < 0.05, Fig. 1e, g). They also showed significant increases in IgG and fold change to two antigenically distinct H7 strains; A/Netherlands/219/2003 (H7N7) (H7.NL03) and A/equine/Kentucky/1a/1975 (H7N7) (H7.Eq75) (Supplemental Fig. 1a, d, e, h, *p* < 0.05). This difference in antibody response does not appear to be due to sampling time differences as there were no obvious differences in the early sera sampling time points.

To examine the breadth of antibody reactivity, we assessed the IgG cross-reactivity against a panel 22 HA proteins from different subtypes encompassing Group 1 (G1) and Group 2 (G2) HA (Supplementary Table 3). The AUC of the ELISA responses was used to reflect the G1 and G2 HA response as well as the totality of the HA-binding antibody response. Similar to the H7-specfic IgG response, early sera from A(H7N9) patients imprinted with A(H3N2/H1N1), showed lower IgG titers against most G1 and G2 HA compared to early sera from P7 and the A(H2N2) imprinting group (Fig. 1i-top panel). IgG-titers increased broadly across subtypes by the late stage of infection (Fig. 1i-middle panel), resulting in significant increases in IgG-AUC (Fig. 1i-lower panel and Fig. 1k), in those imprinted with A(H3N2/H1N1). The geometric mean (GMT) AUC in this group increased by 8.68-fold (95% CI, 2.27 –33.2) to G1 HA and by 9.92-fold (95% CI, 2.6–37.8) to G2 HA (Fig. 1k, Supplemental Table 4). In contrast, the antibody increases were modest in the A(H1N1) and were minimal in A(H2N2)-imprinted group, likely due to the already high cross-reactive IgG-titers detected in the early sera of these patients.

For comparison, we included paired sera from eight severely ill A(H1N1) patients hospitalized in 2018 but that eventually recovered (Supplemental Table 2). Based on their imprinting probabilities, seven were A(H3N2/H1N1) imprinted, and only one was A(H1N1) imprinted. The antibody response after A(H7N9) infection were in stark contrast to the response observed after A(H1N1) infection (Fig. 1l–n), which showed only a modest increase in the GMT AUC of G1 HA by 1.75-fold (95% CI, 0.61–5.01) and of G2 HA by 1.99-fold (95% CI, 0.99–3.99) in A(H3N2/H1N1) group. The broad cross-group IgG response in A(H7N9) patients were also reflected in the antibodies targeting the HA-stalk^13^. A(H7N9) infection increased the GMT of H1 and H3-stalk antibodies, whereas A(H1N1) infection only increased the GMT of H1-stalk antibodies (Supplementary Fig. 2 and Supplementary Table 5), resulting in significant difference in the increase for H3-stalk antibodies between the A(H7N9) and A(H1N1) group (Supplementary Fig. 2c). We assessed the interrelationships among HI, MN, NI, total IgG and stalk-specific IgG antibody titers using correlation analysis (Supplementary Fig. 2d). As expected, a strong and statistically significant positive correlation was observed between HI and MN titers against H7.AN13 (*r* = 0.75, *p* = 0.017), indicating that these two functional assays capture overlapping aspects of the humoral response, likely directed against the immunodominant head domain. Both H1 and H3-stalk antibody levels showed moderate, though not statistically significant, correlations with MN titers (H1-stalk: *r* = 0.57, *p* = 0.065; H3-stalk: *r* = 0.59, *p* = 0.221), suggesting a trend where stalk antibodies contribute to broad neutralization. While stalk antibodies were not strongly linked to HI or HA-specific IgG (H1-stalk: *r* = 0.07; H3 stalk: *r* = −0.18), H1-stalk levels were significantly correlated with NI titers (*r *= 0.58, *p* = 0.048), hinting at overlapping immunological regulation between anti-stalk and anti-NA responses. This further confirms that stalk reactivity represents an immunologically separate axis from head-directed binding and classical neutralization. However, stratifying these responses by early-life imprinting did not reveal statistically significant differences in stalk antibody levels between groups (Supplementary Fig. 2e), suggesting that other factors may dominate the regulation of stalk-specific immunity in this context.

We measured the IgA antibody response in ten patients with sufficient sera against G1 and G2 HA in the A(H7N9) and A(H1N1) cohorts (Supplementary Fig. 3). In contrast to the IgG-response, IgA reactivity was at least 10-fold lower and was more variable across the different imprint groups. A(H7N9) patient had significantly higher IgA antibody titers against G2 HA in early sera compared to A(H1N1) patients (Supplementary Fig. 3e). No clear pattern of birth-imprinting was observed for IgA-responses (Supplementary Fig. 3f). In five A(H7N9) patients with available samples up to 100 days post symptom onset, the H7.AN13 specific IgG and the total IgG AUC remained stable, while the specific IgA titers and total IgA AUC were not sustained over time (Supplementary Fig. 4).

As avidity is an indicator of affinity matured antibodies that contribute to the quality of the antibody response^14^, we measured the IgG avidity against H7, seasonal H1, and H3 proteins using urea-based ELISA in the A(H7N9) patients (Fig. 1o–q and Supplementary Figs. 5, 6). We selected past pandemic strains (H1.BM18, H1.CA09, H3.68) and representative seasonal influenza strains (H1.PR34, H1.BN07, H3.09 and H3.HK14). We derived the avidity index (AI%), measured at 7 M urea concentration which showed the largest binding difference (Supplementary Fig. 5) for each antigen.

We found that the AI profile corresponded with birth-imprint effects. The AI to H7.AN13 was low in the early sera for all, but trended higher for those with high A(H2N2) imprint probability (mean AI(%) = 51.7% vs. 33.1% for A(H1N1), *p* = 0.279, Fig. 1o–q). H7.NL03 showed statistical significance in early sera (*p* = 0.035), reinforcing the A(H2N2) imprinting effect for H7 strains. These patients also had highly IgG avidity to older seasonal H3 (H3.68, H3.09) and H1 (H1.BM18, H1.PR34) but not to more contemporary H1 and H3 strains (H1.BN07, H3.HK14), though most differences represent trends due to small sample sizes (Supplementary Fig. 6). In contrast, those born prior to 1950 and imprinted with A(H1N1) showed low AI to H7 but high AI to older H1 (H1.BM18, H1.PR34 and their antigenic descendent, H1.CA04). These data indicate that while IgG avidity to H7 was generally lower than to seasonal H1 and H3, A(H2N2) imprinted patients displayed relatively high IgG avidity to H7, compared to other patients.

**Fig. 2:**
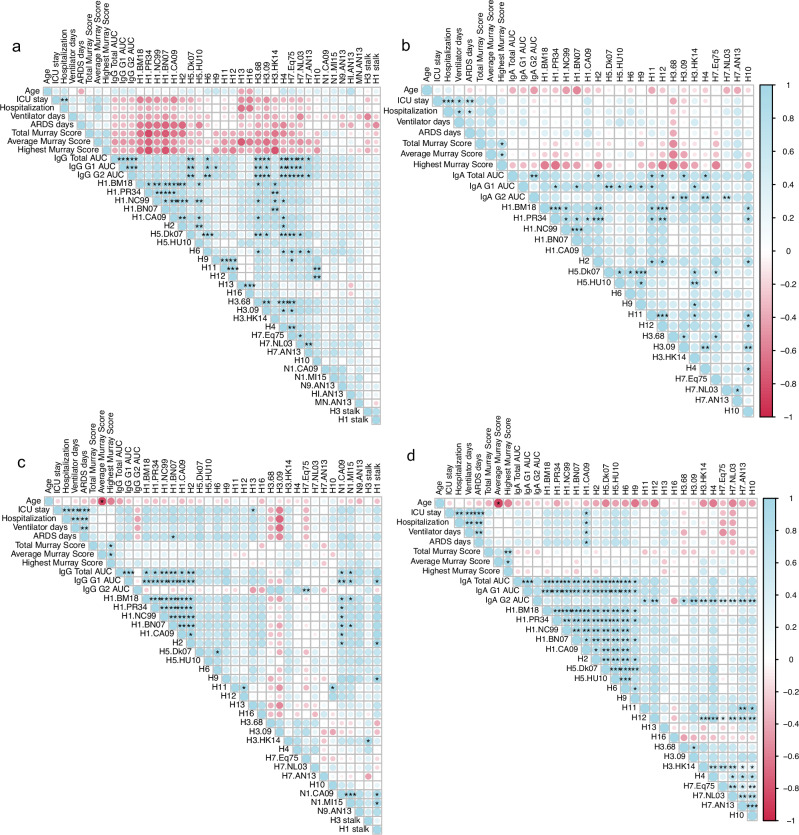
Early antibody landscape and clinical outcome. **a** Correlation matrices for early antibody responses (HA-IgG, NA, HI, MN and HA stalk antibodies) and clinical outcome in A(H7N9) cohort. **b** Correlation matrices for early IgA antibodies and clinical outcome in A(H7N9) cohort. **c** Correlation matrices for early antibody responses (HA-IgG, NA and HA stalk antibodies) and clinical outcome in A(H1N1) cohort. **d** Correlation matrices for early IgA antibodies and clinical outcome in A(H1N1) cohort. Correlations were performed in the corrplot package of R software with Spearman correlation values for each comparison and p-values adjusted by controlling for the false discovery rate using the Benjamini–Hochberg method. *, *p* < 0.05; **, *p* < 0.01; ***, *p* < 0.001; and ****, *p* < 0.0001.

To determine if the antibody profiles were associated with protection, we performed correlation analysis between the early sera antibody profile with the different clinical parameters of infection severity, including the Murray score index^10^ (Fig. 2), which grades the severity of acute respiratory distress syndrome (ARDS). We used three aspects of the Murray score; total, average and highest scores to capture the complexity of infection throughout hospitalization.

In the early sera of A(H7N9) patients, a trend of negative correlation was observed between IgG titers to many seasonal HAs, including H1, H3 subtypes and the cross-reactive H1-stalk and H3-stalk and disease severity, such as ICU stay, hospitalization, and Murray scores (Fig. 2a). A similar pattern of inverse correlation was noted for IgA titers against most seasonal HAs with the highest Murray score (Fig. 2b). While these trends suggest a potential link between pre-existing seasonal influenza immunity and milder H7N9 disease, the correlations did not reach statistical significance. In contrast, following seasonal A(H1N1) infection, the data showed a trend of positive correlation between early IgG and IgA titers against the infecting H1 and N1 antigens and increased disease severity (Fig. 2c, d). Interestingly, an inverse trend was observed for IgG against the H3.68 and H3.09 strains in this cohort. Similarly, IgG avidity in the early sera against H1 (H1.BM18) and H3 (H3.68 and H3.09) showed significant negative associations to adverse clinical outcome in A(H7N9) patients (Supplementary Fig. 7). The consistent trend across multiple seasonal head domains and the conserved stalk region underscores the potential importance of broad, cross-reactive antibody responses in modulating disease outcomes during avian influenza infection.

## Discussion

Using a statistical modeling approach^5^, Gostic et al. proposed that imprint by Group 2 HA, marked by the emergence of A(H3N2) in 1968, protected against severe A(H7N9) disease as reflected by the low numbers of medically-attended cases in this age group. In light of this, we were surprised to see a difference in the antibody profiles of A(H7N9) patients that were likely imprinted at birth to A(H2), a Group 1 HA, compared to the other age groups. Patients in this group showed an antibody response profile that were characteristic of a recall response, with rapid IgG kinetics, minimal IgM and high avidity antibodies^15^. Our data in Fig. 2 suggests that the presence of these avian antibodies could be modestly protective since levels of anti-H2, H5 and H4 were negatively correlated with clinical severity after A(H7N9) infections.

One probable explanation is that H2 and H7 may share conserved avian epitopes that were preferentially recalled despite being in a different phylogroup. Our group had previously demonstrated that A(H2N2) imprinting also conferred durable cross-reactive and protective NA antibodies to group 2 avian N2 and N3^16^, suggesting that early life exposure by avian-origin viruses may confer lifelong protective antibodies. This challenges the simple HA group-based dichotomy and suggests that early-life exposure to avian-origin viruses can establish a uniquely broad and lifelong protective antibody repertoire. To investigate this at a population level, we re-analyzed the case data from Supplementary Tables 3 and 4 of Gostic et al.^5^, grouping individuals by birth year to infer their most likely imprinting subtype (Supplemental Fig. 8). This analysis reveals a critical hierarchy of protection against severe H7N9 that is obscured by the broad “group 1” classification. The A(H2N2)-imprinted cohorts (1950-1957 and 1958-1968) exhibited a substantially lower susceptibility to fatal A(H7N9) disease compared to the earlier cohort imprinted primarily with A(H1N1) (Before 1950), with fatality rates dropping from 28.1% to 21.4% and 13.4%, respectively. This trend, while less pronounced than the 10.8% fatality in the A(H3N2)-imprinted (post-1968) group, provides strong epidemiological support for our immunological finding: A(H2N2) imprinting offers a unique advantage in mitigating disease severity. Our data therefore updates Gostic et al.’s model by reclassifying the older cohorts born prior to 1968, to individuals imprinted with A(H1N1) as being more susceptible to severe outcomes and those imprinted with A(H2N2) as those who are more likely primed for a potent, modulatory response after H7N9 virus infection. P7 can be used to reflect this hierarchy. P7 with a high likelihood of being imprinted only with the 1968 avian H3 or a similar variant, had high IgG to diverse HA (including H7.AN13) but did not elicit as high titers of high avidity antibodies to H7 as those imprinted with A(H2N2).

Similar to previous findings^17^, broad IgG-cross-reactivity was observed after A(H7N9) infection in a predominantly older cohort of patients hospitalized during the first wave of A(H7N9) outbreak. In our study, this expanded breadth of cross-reactivity seemed to be independent of the imprint history and infection severity since severely ill A(H1N1) patients did not show such broad response. The breadth and magnitude of the antibody response observed in our study may have been boosted by the presence of preexisting, non-neutralizing immunity and/or A(H7N9) infection may be better than A(H1N1), by way of greater viral burden during infection, at eliciting broad cross-group antibodies in humans.

HAI and NAI antibody titers are currently well-accepted correlates of immune protection against influenza^18^. Here, we observed a consistent trend wherein early IgG titers against a broad range of seasonal influenza HAs were inversely correlated with disease severity, suggesting a potential protective role for cross-reactive immunity. As influenza vaccination uptake is relatively low in China, one possible explanation is that these patients were recently infected, therefore maintaining relatively higher levels of cross-protective immunity that may include T cell responses. On the other hand, high titers of early IgG and IgA against G1 strains were correlated to poorer clinical outcomes following seasonal A(H1N1) infection. It is possible that the severe A(H1N1) patients had low pre-existing titers to begin with and may have experienced a hyperactivation of the immune response, although not to the levels seen in the A(H7N9) infections, or are experiencing other immune-mediated pathology resulting from a dysregulated antibody production^19^.

As our study only investigated the humoral immunity, another potential source of heterologous protection could be conferred by pre-existing cross-reactive CD8⁺ T-cell (CTL) immunity. Memory CTLs, generated from prior exposure to seasonal influenza strains can recognize conserved internal epitopes (e.g., from NP and M1 proteins) on H7N9-infected cells^20^. This pre-existing memory pool enables the rapid recruitment, clonal expansion, and cytotoxic activity of virus-specific T cells, leading to early viral control and reduced disease severity, as evidenced by patients who recover quickly showing robust, immediate CD8⁺ T-cell responses^11^. Conversely, the absence or inefficiency of such cross-reactive immunity, which can be influenced by antigenic variability, HLA diversity, and ethnic background, results in delayed, minimal, or dysfunctional T-cell responses (characterized by a persistent CD38⁺HLA-DR⁺PD-1⁺ exhausted phenotype), contributing to poor clinical outcomes and fatal disease^11,21^. However, sequence conservation does not always predict cross-protective CTL immunity^22^, and how the different imprint history regulates the T cell immunity is still unclear.

This study had several limitations. The overall sample size was inherently limited by the nature of A(H7N9) cases, and this constraint was exacerbated upon stratification by variables such as age, infection wave, or early-life immune imprinting group. The particularly small subgroups within the imprinting analysis (e.g., A(H2N2), A(H1N1)) resulted in statistically underpowered correlation matrices, precluding robust inference regarding differences in clinical trajectories or immune correlates across these strata. Furthermore, the retrospective, observational nature of the study means associations cannot establish causality, and cohorts such as ‘Before 1950’ may be confounded by older age and comorbidities. It is difficult to ascertain accurately the imprint history, particularly those born on the cusp of the pandemic transitions. This could have contributed to some variability in the responses observed.

In conclusion, we demonstrated that there was a birth-imprinting-specific component to the kinetics, magnitude and avidity of the antibody response after A(H7N9) infection that was not evident in seasonal A(H1N1) infections. Additionally, seasonal influenza antibodies were able to reduce disease severity in these patients, suggesting that annual influenza vaccination maybe a viable strategy to mitigate the risk of severe infections in the event of an avian influenza virus outbreak^23^.

## Supplementary information

Supplementary Material File

Supplementary Data 1

Supplementary Data 2

Description of Additional Supplementary Files

## Data Availability

Source data for all figures are provided in the following files: Fig.1.xlsx (Fig. 1), Fig.2.xlsx (Fig. 2), and Supplementary Figs. 1–7.xlsx (Supplementary Figs. 1–7). All files are also available on figshare (10.6084/m9.figshare.31420415)^24^.
