# Supplementary material for: Birth imprinting effects on the antibody responses of H7N9 patients from 2013-2018 in China
Source: Commun Med (Lond). 2026 Apr 9;6:327. doi: 10.1038/s43856-026-01554-1 (PMC13237065; doi:10.1038/s43856-026-01554-1)
Supplement: Supplementary file 1 — Supplementary Material File [file 43856_2026_1554_MOESM1_ESM.pdf]

Supplemental Figures and Tables

Supplemental Fig. 1 | H7-specific antibody response in A(H7N9) cases from different groups.
Supplemental Fig. 2 | HA stalk antibody profile.
Supplemental Fig. 3 | Heatmap of IgA-cross-reactivity against Group 1 and Group 2 HA in A(H7N9) and A(H1N1) patients.
Supplemental Fig. 4 | Kinetics and longevity of IgG and IgA antibody cross-reactivity.
Supplemental Fig. 5 | IgG antibody avidity after A(H7N9) infection.
Supplemental Fig. 6 | HA imprinting pattern affect the IgG antibody avidity after A(H7N9) infection.
Supplemental Fig. 7 | Early IgG antibody avidity and clinical outcome.
Supplemental Fig. 8 | Case counts and clinical outcome distribution by birth year cohort following H7N9 infection (Data from Gostic et al.).
Supplemental Table 1. Characteristics of A(H7N9) patients in the study.
Supplemental Table 2. Characteristics of A(H1N1) patients in the study.
Supplemental Table 3. List of recombinant HA protein used in the ELISA.
Supplemental Table 4. Geometric mean fold change of IgG area under curve (AUC) between early and late and the corresponding 95% Confidence Intervals.
Supplemental Table 5. Geometric mean HA stalk IgG antibody titers and the corresponding 95% Confidence Intervals.

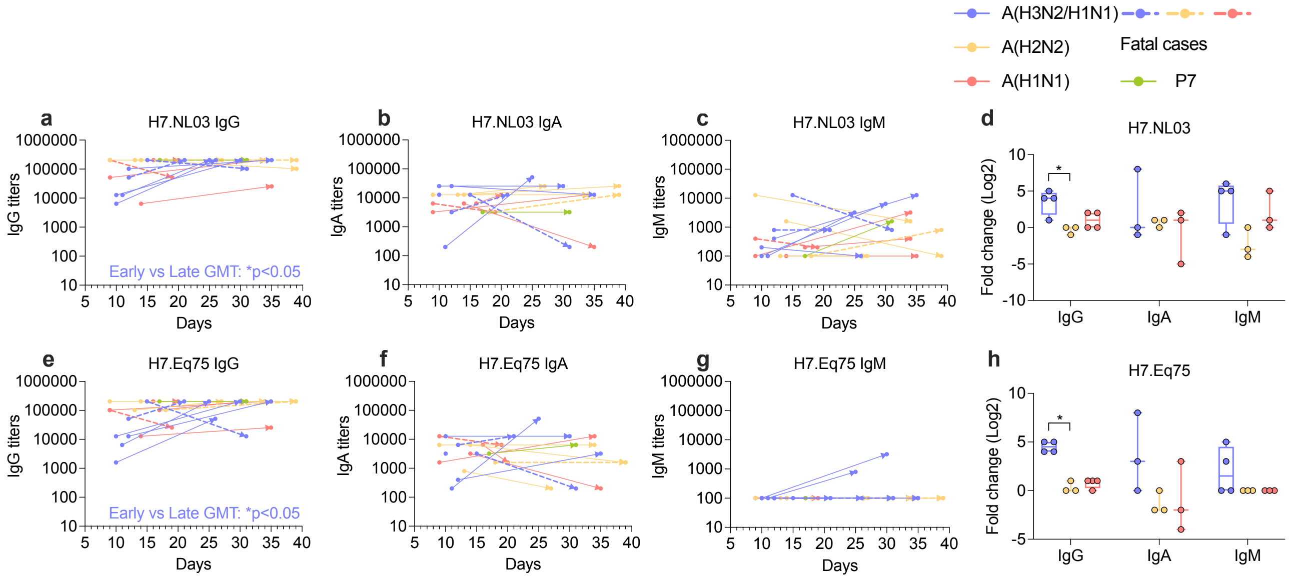

Supplemental Fig. 1 | H7-specific antibody response in A(H7N9) cases from different groups. a-h H7-specific IgG, IgA and IgM titers against A/Netherlands/219/03 (H7N9) (H7.NL03) and A/equine/Kentucky/1a/1975 (H7N9) (H7.Eq75) in early and late sera of different groups. Fold change of antibody titers of H7-specific IgG, IgA and IgM between early and late in three groups are shown. Statistical significance between early and late antibody titers from each group in (a-c, e-g) were analyzed by paired t-test. Differences among three groups in (d and h) were determined by non-parametric Kruskal-Wallis Test, then the pairwise comparisons with significant *p*-values, *, $p<0.05$; **, $p<0.01$; ***, $p<0.001$; and ****, $p<0.0001$.

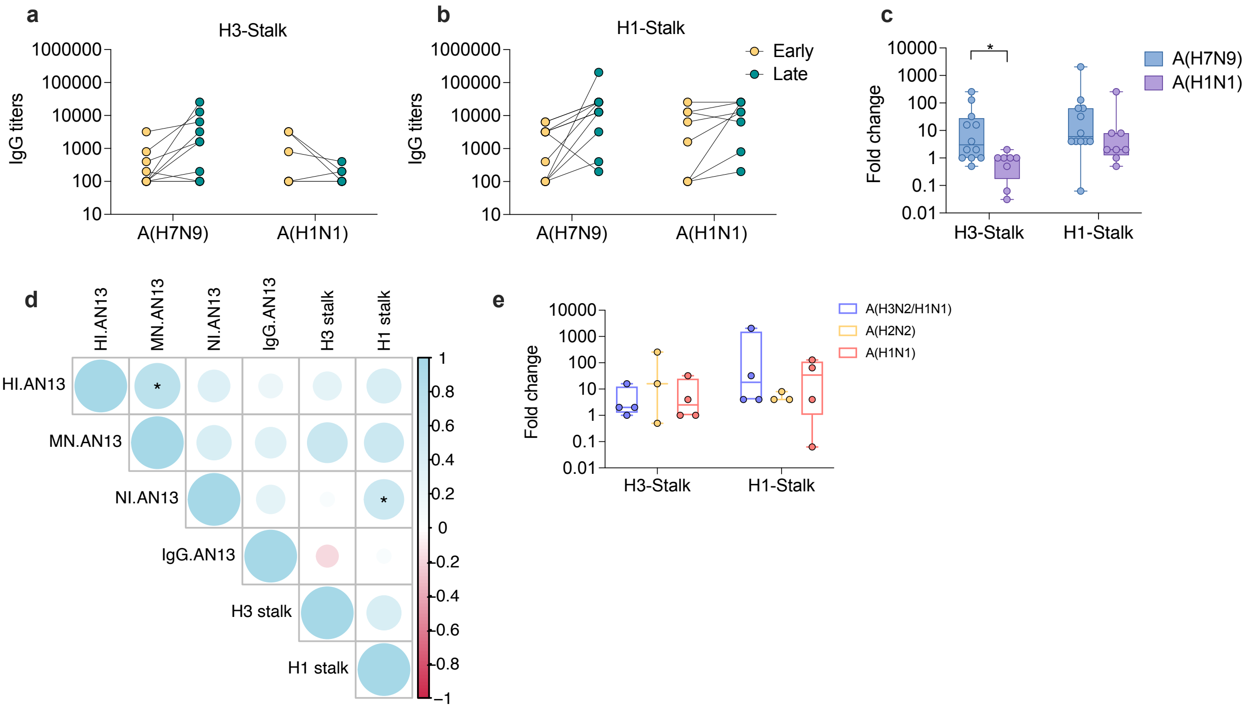

Supplemental Fig. 2 | HA stalk antibody profile. a-b Anti-HA stalk antibodies of individuals from A(H7N9) and A(H1N1) cohorts, with H1 stalk of pH1HA10-Foldon (designed from A/California/04/2009 (H1N1)) or H3 stalk of HK68-H3-SI (designed from A/Hong Kong/1/1968 (H3N2)) by ELISA. **c** Fold change of anti-HA stalk antibodies between early and late serum samples. Statistical significance between A(H7N9) and A(H1N1) in (c) were analyzed by the Mann-Whitney U test, *, $p<0.05$; **, $p<0.01$; ***, $p<0.001$; and ****, $p<0.0001$.

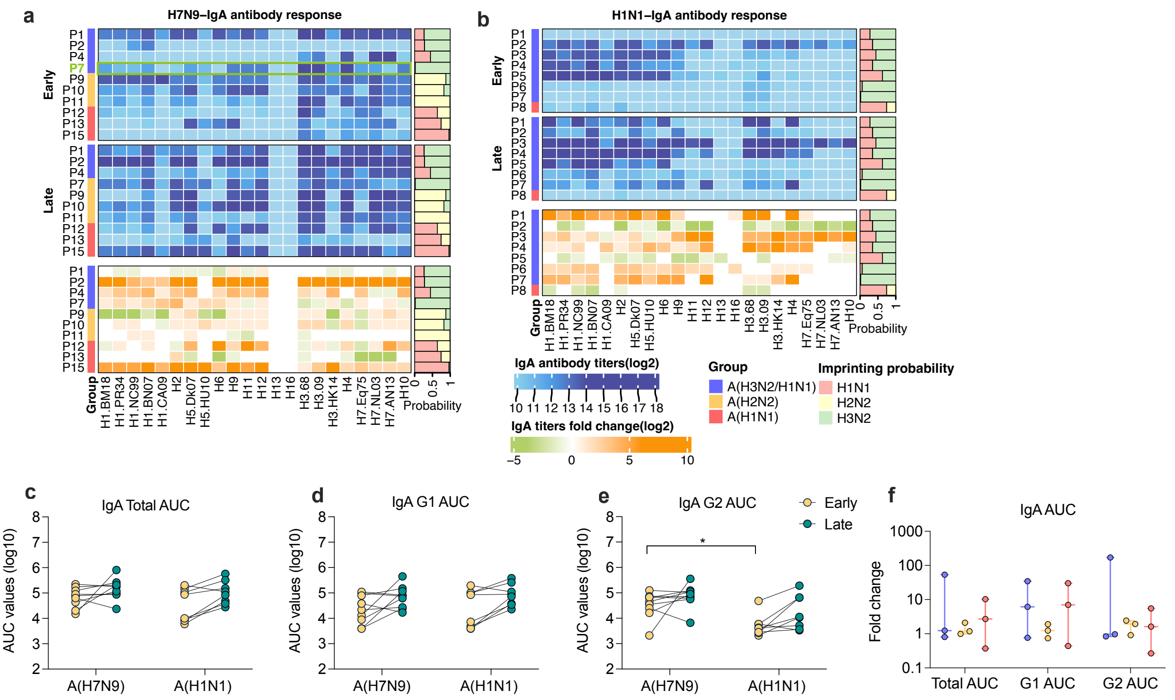

Supplemental Fig. 3 | Heatmap of IgA-cross-reactivity against Group 1 and Group 2 HA in A(H7N9) and A(H1N1) patients. a-b Heatmap of IgA titers in different groups of A(H7N9) and A(H1N1) patients against both Group 1 (G1) and Group 2 (G2) HA. Serum from early (top panel) and late (middle panel) stages of A(H7N9) (A(H3N2/H1N1), n=4; A(H2N2), n=3; A(H1N1), n=3) and A(H1N1) (A(H3N2/H1N1), n=7; A(H1N1), n=1) patients were measured by ELISA. The end point titers were log_2 transform and used to generate heatmaps using the "ComplexHeatmap" package in R (version 411 4.0.5). Fold changes of antibody titer between early and late sera against G1 and G2 HA were shown in the lower panel. **c-e** AUC values for the early and late stage of A(H7N9) and A(H1N1) patients against IgA total, G1 and G2 HA. **g** Fold change in IgA total HA, G1 and G2 area under curve (AUC) values between early and late sera of A(H7N9) patients from the different imprint groups. Differences between A(H7N9) and A(H1N1) in (c-f) were analyzed by the two-sided Wilcoxon signed rank test, *, *p*<0.05; **, *p*<0.01; ***, *p*<0.001; and ****, *p*<0.0001.

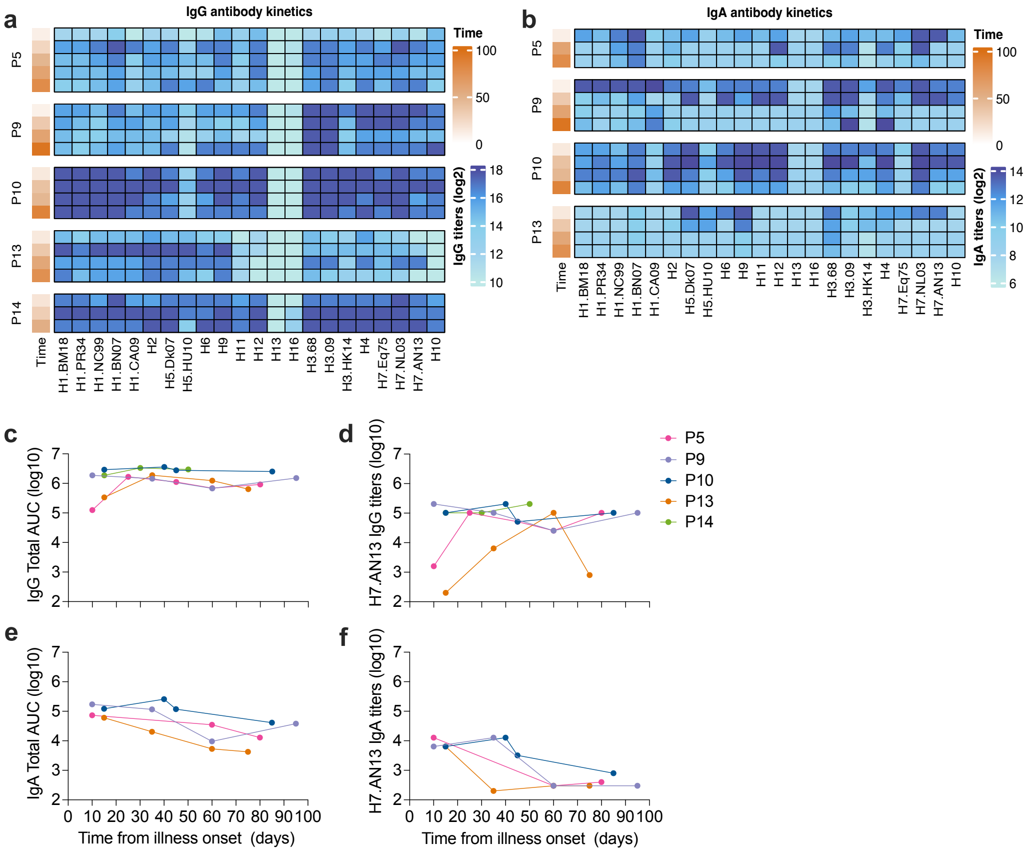

Supplemental Fig. 4 | Kinetics and longevity of IgG and IgA antibody cross-reactivity. a-b Heatmap analysis of IgG and IgA antibody titers against G1 and G2 HA over time. **c-f** Kinetics and longevity of IgG Total AUC, IgG titers against AN13, IgA Total AUC and IgA titers against H7.AN13.

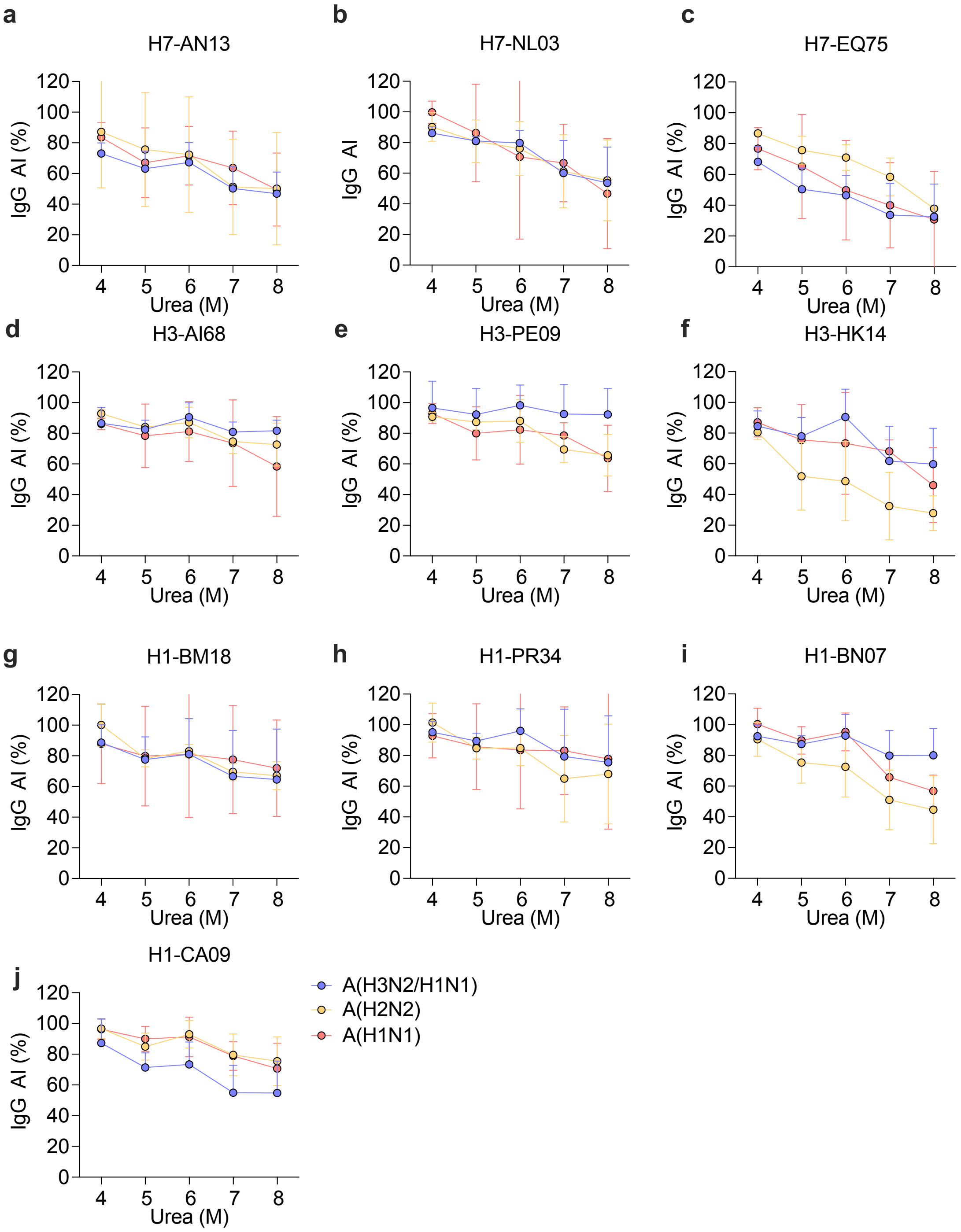

Supplemental Fig. 5 | IgG antibody avidity after A(H7N9) infection. IgG avidity after different concentration (4-8M) urea wash in the late stage from A(H7N9) infection of different groups. The avidity index (AI%) after urea wash against H7 antigens (**a-c**), A/Anhui/1/2013 (H7.AN13), A/Netherlands/219/03 (H7.NL03) and A/equine/Kentucky/1a/1975 (H7.Eq75), H3 antigens (**d-f**), A/Aichi/2/1968 (H3.68), A/Perth/16/2009 (H3.09) and A/HongKong/4801/2014 (H3.HK14), H1 antigens (**g-j**), A/Brisbane/59/2007 (H1.BN07), A/Brevig Mission/1/1918 (H1.BM18) and A/California/04/2009 (H1.CA09) were determined by urea-based ELISA.

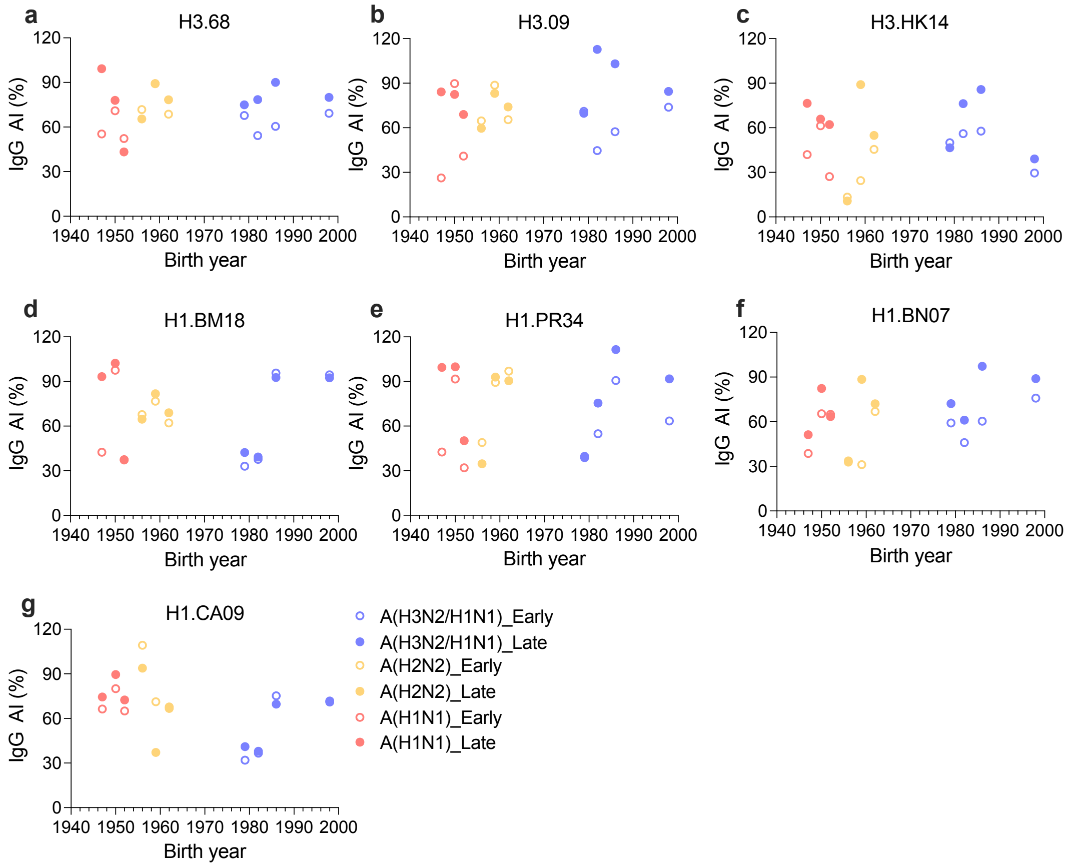

Supplemental Fig. 6 | HA imprinting pattern affect the IgG antibody avidity after A(H7N9) infection. IgG avidity after 7M urea wash in the early and late stage of A(H7N9) infection based on the year of birth were shown. The avidity index (AI%) after 7M urea wash against H3 antigens (**a-c**), A/Aichi/2/1968 (H3.68), A/Perth/16/2009 (H3.09) and A/HongKong/4801/2014 (H3.HK14), H1 antigens (**d-g**), A/Brisbane/59/2007 (H1.BN07), A/Brevig Mission/1/1918 (H1.BM18) and A/California/04/2009 (H1.CA09) were determined by urea-based ELISA.

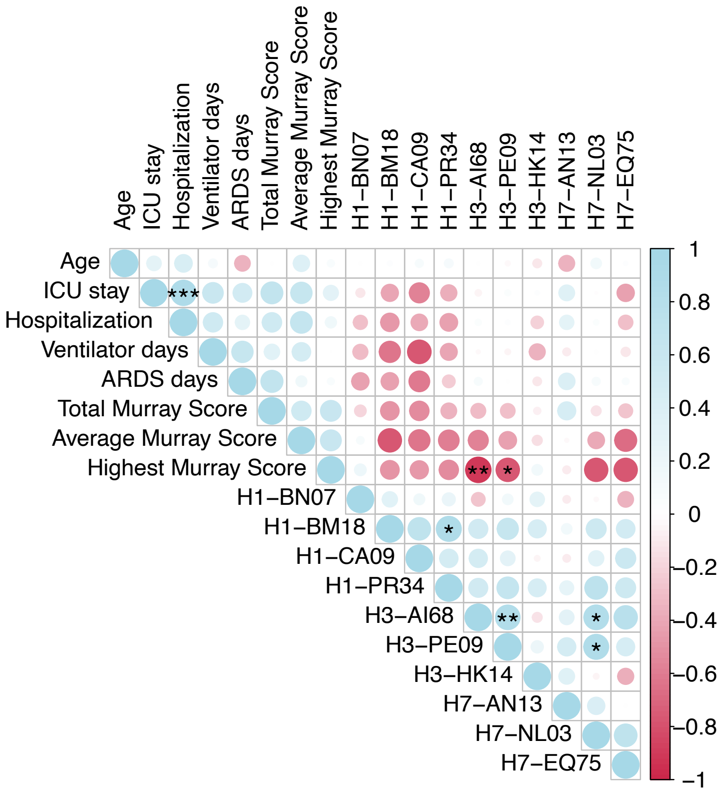

Supplemental Fig. 7 | Early IgG antibody avidity and clinical outcome. Correlation matrix for early IgG antibody avidity and clinical outcome in A(H7N9) cohorts. Correlations were performed in the corrplot package of R software with Spearman correlation values for each comparison and p-values adjusted by controlling for the False Discovery Rate using the Benjamini-Hochberg method. *, $p<0.05$; **, $p<0.01$; ***, $p<0.001$; and ****, $p<0.0001$.

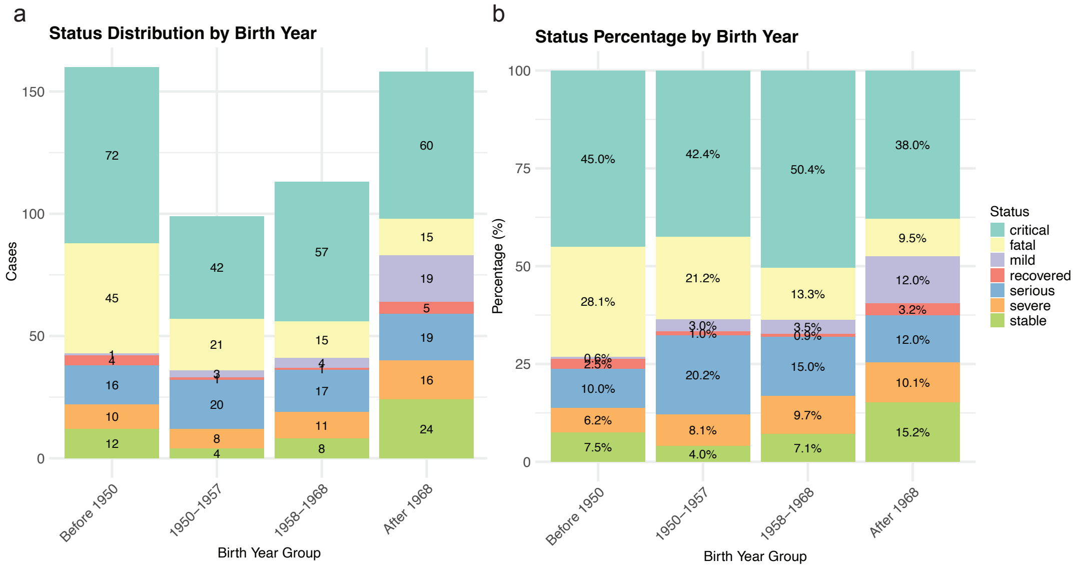

Supplemental Fig. 8 | Case counts and clinical outcome distribution by birth year cohort following H7N9 infection (Data from Gostic et al.).
(a) The total number of cases and across four birth year groups (Before 1950, 1950-1957, 1958-1968, After 1968), categorized by clinical status (critical, fatal, mild, recovered, serious, severe, stable) and (b) the proportional distribution of these statuses within each group.

Supplemental Table 1. Characteristics of A(H7N9) patients in the study.

Patient no.	Group	Gender	Early	Late	Sampling year	Infection wave	Clinical Outcome	Duration of hospitalization (days)	ICU stay (days)
P1	A(H3N2/H1N1)	F	10	30	2015	3rd	Discharged	25	20
P2	A(H3N2/H1N1)	M	11	25	2014	2nd	Discharged	20	15
P3	A(H3N2/H1N1)	F	12	21	2014	2nd	Deceased	86	84
P4	A(H3N2/H1N1)	M	12	35	2015	3rd	Discharged	39	24
P5	A(H3N2/H1N1)	F	10	26	2014	2nd	Discharged	81	61
P6	A(H3N2/H1N1)	M	15	31	2013	1st	Deceased	26	24
P7	A(H3N2/H1N1)	M	17	31	2014	2nd	Discharged	21	16
P8	A(H2N2)	M	18	34	2017	5th	Deceased	36	36
P9	A(H2N2)	F	9	39	2018	5th	Discharged	101	57
P10	A(H2N2)	F	14	39	2013	1st	Discharged	38	24
P11	A(H2N2)	M	13	27	2014	2nd	Discharged	26	12
P12	A(H1N1)	M	16	20	2017	5th	Discharged	22	18
P13	A(H1N1)	M	14	35	2014	2nd	Discharged	53	35
P14	A(H1N1)	M	17	31	2017	5th	Discharged	65	38
P15	A(H1N1)	F	9	34	2013	1st	Discharged	44	38
P16	A(H1N1)	F	9	19	2017	5th	Deceased	26	20

Supplemental Table 2. Characteristics of A(H1N1) patients in the study.

Patient no.	Group	Gender	Early	Late	Sampling year	Clinical Outcome	Duration of hospitalization (days)	ICU stay (days)
P1	A(H3N2/H1N1)	M	8	22	2018	Discharged	22	20
P2	A(H3N2/H1N1)	F	11	20	2018	Discharged	21	17
P3	A(H3N2/H1N1)	F	11	15	2018	Discharged	50	50
P4	A(H3N2/H1N1)	F	13	46	2018	Discharged	39	34
P5	A(H3N2/H1N1)	F	19	31	2018	Discharged	72	72
P6	A(H3N2/H1N1)	M	10	28	2018	Discharged	25	22
P7	A(H3N2/H1N1)	F	9	22	2018	Discharged	38	18
P8	A(H1N1)	M	7	13	2018	Discharged	9	0

Supplemental Table 3. List of recombinant HA protein used in the ELISA.

No.	Group	Subtype	Strain	Catalog Number	Abbreviation
1	1	H1N1	A/Brevig Mission/1/1918	11068-V08H	H1.BM18
2	1	H1N1	A/Puerto Rico/8/34	11684-V08H	H1.PR34
3	1	H1N1	A/New Caledonia/20/1999	11683-V08H	H1.NC99
4	1	H1N1	A/Brisbane/59/2007	11052-V08H	H1.BN07
5	1	H1N1	A/California/07/2009	11085-V08H	H1.CA09
6	1	H2N2	A/Japan/305/1957	11088-V08H	H2
7	1	H5N3	A/duck/Hokkaido/167/2007	11696-V08H	H5.Dk07
8	1	H5N1	A/Hubei/1/2010	40015-V08H	H5.HU10
9	1	H6N4	A/chicken/Hong Kong/17/1977	40027-V08H	H6
10	1	H9N2	A/Hong Kong/1073/99	11229-V08H	H9
11	1	H11N2	A/duck/Yangzhou/906/2002	11705-V08H	H11
12	1	H12N5	A/green-winged teal/ALB/199/1991	11718-V08H	H12
13	1	H13N8	A/black-headed gull/Netherlands/1/00	11721-V08H	H13
14	1	H16N3	A/black-headed gull/Sweden/5/99	11711-V08H	H16
15	2	H3N2	A/Aichi/2/1968	11707-V08H	H3.68
16	2	H3N2	A/Perth/16/2009	40043-V08H	H3.09
17	2	H3N2	A/HongKong/4801/2014	40555-V08B	H3.HK14
18	2	H4N6	A/mallard/Ohio/657/2002	11714-V08H	H4
19	2	H7N9	A/Anhui/1/2013	40103-V08H	H7.AN13
20	2	H7N7	A/Netherlands/219/2003	11082-V08B	H7.NL03
21	2	H7N7	A/equine/Kentucky/1a/1975	40171-V08B	H7.Eq75
22	2	H10N3	A/duck/Hong Kong/786/1979	11693-V08H	H10

Supplemental Table 4. Geometric mean fold change of IgG area under curve (AUC) between early and late and the corresponding 95% Confidence Intervals.

Infection	Group	IgG Total AUC	IgG G1 AUC	IgG G2 AUC
A(H7N9)	A(H3N2/H1N1)	9.16 (2.52, 33.3)	8.68 (2.27, 33.2)	9.92 (2.6, 37.8)
	A(H2N2)	1.27 (0.62, 2.63)	1.55 (0.32, 7.65)	1.08 (0.84, 1.4)
	A(H1N1)	2.43 (0.73, 8.12)	2.7 (0.682, 10.7)	1.64 (0.77, 3.53)
A(H1N1)	A(H3N2/H1N1)	1.99 (0.99, 3.99)	1.75 (0.61, 5.01)	1.99 (0.99, 3.99)

Supplemental Table 5. Geometric mean HA stalk IgG antibody titers and the corresponding 95% Confidence Intervals.

Infection	H3-Stalk			H1-Stalk		
	Early	Late	Fold change	Early	Late	Fold change
A(H7N9)	189 (92, 386)	1131 (282, 4526)	5.99 (1.62, 22.15)	800 (259, 2468)	10159 (2994, 34475)	12.70 (2.49, 64.81)
A(H1N1)	308 (80, 1187)	141 (91, 219)	0.46 (0.13, 1.61)	1600 (210, 12197)	6400 (1403, 29188)	4.00 (0.80, 20.00)
