# Supplementary material for: Birth imprinting effects on the antibody responses of H7N9 patients from 2013-2018 in China
Source: Commun Med (Lond). 2026 Apr 9;6:327. doi: 10.1038/s43856-026-01554-1 (PMC13237065; doi:10.1038/s43856-026-01554-1)
Supplement: Supplementary file 4 — Description of Additional Supplementary Files [file 43856_2026_1554_MOESM4_ESM.docx]

**Description of Additional Supplementary Files**

File name: Supplementary Data 1-2

Description: The calculation of the Murray score at different days after symptom onset in A(H7N9) and A(H1N1) patients
